# Effects of nicotine on regional blood flow in the olfactory bulb in response to olfactory nerve stimulation

**DOI:** 10.1186/s12576-020-00758-x

**Published:** 2020-06-10

**Authors:** Sae Uchida, Fusako Kagitani

**Affiliations:** Department of Autonomic Neuroscience, Tokyo Metropolitan Institute of Gerontology, 35-2 Sakaecho, Itabashi-ku, Tokyo, 173-0015 Japan

**Keywords:** Olfactory bulb, Olfactory nerve stimulation, Regional cerebral blood flow, Nicotinic acetylcholine receptor, Rat

## Abstract

This study examined the effect of olfactory nerve stimulation on regional cerebral blood flow and assessed the effect of intravenous nicotine administration on this response in anesthetized rats. Regional cerebral blood flow was measured with laser Doppler flowmetry or laser speckle contrast imaging. Unilateral olfactory nerve stimulation for 5 s produced current (≥ 100 μA) and frequency-dependent (≥ 5 Hz) increases in blood flow in the olfactory bulb ipsilateral to the stimulus. The increased olfactory bulb blood flow peaked at 30 ± 7% using stimulus parameters of 300 μA and 20 Hz. Nerve stimulation did not change frontal cortical blood flow or mean arterial pressure. The intravenous injection of nicotine (30 μg/kg) augmented the olfactory bulb blood flow response to nerve stimulation (20 Hz, 300 μA) by approximately 1.5-fold (60-s area after the stimulation). These results indicate that olfactory nerve stimulation increases olfactory bulb blood flow, and the response is potentiated by the activation of nicotinic cholinergic transmission.

## Introduction

The cholinergic neurons originating in the basal forebrain send projections to the neocortex, hippocampus, and olfactory bulb that contribute to cognition, memory, and olfactory function, respectively [1–3]. Our research group previously found in anesthetized rats that activation of basal forebrain cholinergic neurons produces an increase in regional blood flow in the neocortex [4–7] and hippocampus [8], whereas it does not influence blood flow in the olfactory bulb [9]. Blood flow in the olfactory bulb is reportedly increased by odor stimulation in association with neuronal activities [10–14]. Our recent study confirmed that odor stimulation (5% amyl acetate) produces an increase in blood flow in the olfactory bulb in anesthetized rats [15].

The olfactory nerve transmits information about the sense of smell from the olfactory epithelium to the olfactory bulb. Rodent studies have reported that the olfactory nerve increases its firing frequencies depending on the odor concentration [16–18]. Therefore, we can assume that the olfactory nerve is involved in the odor stimulation-induced increase in blood flow in the olfactory bulb. Multiple in vivo studies have observed blood flow responses in the olfactory bulb evoked by natural olfactory stimulation of odor inhalation and examined the mechanism of neurovascular couplings [19–21]. High-concentration odor inhalation or sustained odorant inhalation can easily produce peripheral adaptation, i.e., a decline in response by olfactory receptor neurons [20]. To improve our neurophysiological understandings of olfactory bulb blood flow response to olfactory input, it is necessary to study the effect of sensory (olfactory) nerve stimulation, in addition to natural olfactory stimulation of odor inhalation. However, no study has examined the effect of olfactory nerve stimulation on olfactory bulb blood flow. Therefore, the present study first aimed to clarify whether olfactory nerve stimulation increases blood flow in the olfactory bulb. By varying the stimulus frequencies of olfactory nerve electrical stimulation, we could quantify the strength of odor stimulation. In this study, electrical stimulation of the olfactory nerve using various stimulus parameters on olfactory bulb blood flow was tested. For comparison, blood flow in the frontal cortex and systemic arterial pressure were measured simultaneously.

Our previous study demonstrated that the increased response of olfactory bulb blood flow to odor stimulation is potentiated by the intravenous injection of nicotine (30 μg/kg) [15]. This potentiation effect of nicotine was mediated by the activation of α4β2-like nicotinic acetylcholine receptors (nAChRs) in the brain [15]. In the present study, we also examined whether the intravenous injection of nicotine potentiated the olfactory bulb blood flow response.

## Methods

### Experimental animals

The experiments were performed on 10 male adult Wistar (*n* = 5) or Fisher (*n* = 5) rats (body weight, 300–440 g; 4–8 months old). All data from different strains were combined because there were essentially no differences in their responses. The study was conducted in accordance with the Guidelines for Proper Conduct of Animal Experiments (established by the Science Council of Japan in 2006) and was approved by the animal care and use committee of Tokyo Metropolitan Institute of Gerontology.

### General surgery and anesthesia

The rats were anesthetized with urethane (1.2–1.4 g/kg, subcutaneously), after the initial inhalation of 4.2% sevoflurane for approximately 5 min. Respiration was maintained using an artificial respirator (model 683, Harvard, Massachusetts, USA) through a tracheal cannula. The end-tidal CO_2_ concentration was maintained at 3.0–4.0% via monitoring with a respiratory gas monitor (Microcap, Oridion Medical, Jerusalem, Israel). Arterial blood pressure was measured through a catheter inserted into a femoral artery with a pressure transducer (TP-400T, Nihon Kohden, Tokyo, Japan). Body temperature was measured rectally and continuously using a thermistor and maintained at approximately 37.5 °C using a body temperature control system (ATB-1100, Nihon Kohden). The depth of anesthesia was adjusted with additional urethane doses (100 mg/kg, i.v. via a catheter inserted into a femoral vein) when necessary and by monitoring body movement, blood pressure stability, and respiratory movement.

### Measurement of regional blood flow in the olfactory bulb and cortex

The animals were mounted on a stereotaxic instrument (SR-5R-HT, Narishige, Tokyo, Japan) in a prone position. Regional cerebral blood flow was measured using laser Doppler flowmetry (in four rats) or laser speckle contrast imaging (in six rats), as described previously [9, 15, 22–24]. We used either laser Doppler flowmetry or laser speckle contrast imaging, depending on the purpose of each experiment. To analyze olfactory blood flow responses at different intensities and frequencies of olfactory nerve stimulation, we used mainly laser Doppler flowmetry because of technical convenience. To analyze spatiotemporal changes in cerebral blood flow following olfactory nerve stimulation, we used laser speckle contrast imaging which provide a wide field of view. To analyze pharmacological effects, we used both devices in four animals each.

For laser Doppler flowmetry, two recording probes (diameter, 0.8 mm) of the laser Doppler flowmeter (ALF 21D, Advance, Tokyo, Japan) were placed, avoiding the visible blood vessels, on the dorsal surface of the unilateral olfactory bulb (AP = + 7.0–8.0 mm from bregma, *L* = 0.6–1.6 mm to the midline), and the frontal cortex (AP = + 1.0–4.0 mm, *L* = 1.0–4.0 mm) [25, 26] following a partial craniotomy. The flowmeter probes were fixed with balancing holders (ALF-B, Advance).

For laser speckle contrast imaging, after craniotomy, the surface of the brain was covered with mineral oil, followed by a glass coverslip. The laser speckle contrast imaging device was then fixed, and the zoom was adjusted to cover the dorsal surface of the brain from the most anterior part of the olfactory bulb to the frontal cortex. Laser speckle contrast imaging was performed using a Moor full-field perfusion imaging device consisting of an infrared laser diode (785 nm wavelength) and a CCD camera (Moor Instruments, Devon, UK). The viewing field covered approximately 108 mm^2^ (12 mm × 9 mm) with a matrix of 150 × 116 pixels, providing an approximate resolution of 79 μm per pixel. The images were sampled at 25 Hz.

To analyze spatial changes in blood flow, the acquired images were further averaged over 1-s time bins. The baseline image just before olfactory nerve stimulation (− 1 to 0 s) was then subtracted from the other images to assess the relative blood flow changes. To quantify the temporal blood flow changes (in arbitrary units), time courses were extracted in three regions of interest (ROIs) depicted by 1.0-mm-diameter circles positioned bilaterally, avoiding the visible blood vessels, in the area of the olfactory bulb (AP = + 7.0–8.0 mm from bregma, *L* = 0.6–1.6 mm to the midline), and the frontal cortex (AP = + 1.0–4.0 mm, *L* = 1.0–4.0 mm) ipsilateral to the side of olfactory nerve stimulation.

### Stimulation of the olfactory nerve

The unilateral olfactory nerve was electrically stimulated as described previously [27]. For stimulation, an additional craniotomy was performed over the olfactory nerve just caudal to the cribriform plate [27]. A coaxial metal electrode with an outer diameter of 0.2 mm was stereotaxically inserted into the olfactory nerve bundle approximately at 4 mm posterior to the nasofrontal suture and approximately 0.8 mm lateral to the midline. Electrical stimulation of the olfactory nerve was performed using a stimulator (SEN-3301, Nihon Kohden) and stimulus isolation unit (SS-202J, Nihon Kohden). Repetitive electrical square pulse stimuli of 0.5 ms in width with varying intensities (20–400 μA) and frequencies (0.5–200 Hz) were applied for 5 s. To examine the relationships between the intensities and frequencies of electrical stimulation of the olfactory nerve and magnitudes of blood flow responses in the olfactory bulb, the stimuli were applied in the order of low to high intensities or frequencies, and the minimum interval between stimuli was 1 min. To examine the effect of nicotine on the olfactory nerve stimulation-induced blood flow response in the olfactory bulb, the parameters of electrical stimulation were set at an intensity of 300 μA with three different frequencies (2, 20, and 100 Hz). The order of these three different frequencies was random, and the minimum interval between stimuli was 3 min.

### Drug administration

(−) Nicotine (Tokyo Kasei Kogyo, Tokyo, Japan) was diluted in saline to final concentration of 30 μg/kg body weight (calculated as the free base). Nicotine was injected slowly (duration of approximately 1 min) through a femoral vein. We chose a dose of 30 μg/kg nicotine because our previous report found that this dose was optimal for stimulating nicotinic cholinergic receptors in the brain parenchyma without causing marked changes in systemic arterial pressure [28] and was effective for enhancing olfactory bulb blood flow responses to odor [15].

### Data collection and statistical analysis

The obtained analog signals of regional blood flow and systemic arterial pressure were recorded on a PC using an A/D converter (Micro 1401 mkII, Cambridge Electronic Design, Cambridge, UK) with Spike 2 software (Spike 2, Cambridge Electronic Design) for offline analyses.

All values are presented as the mean ± SEM. Changes of regional blood flow and mean arterial pressure evoked by olfactory nerve stimulation were assessed by one-way repeated-measures ANOVA followed by Dunnett’s multiple comparison test. The comparison of changes in regional blood flow to olfactory nerve stimulation before and after nicotine injection were conducted using two-way repeated-measures ANOVA followed by Bonferroni’s multiple comparison test or Wilcoxon’s matched-pairs signed-rank test. *p* < 0.05 was considered statistically significant.

## Results

### Blood flow response to olfactory nerve stimulation in the olfactory bulb

**Fig. 1:**
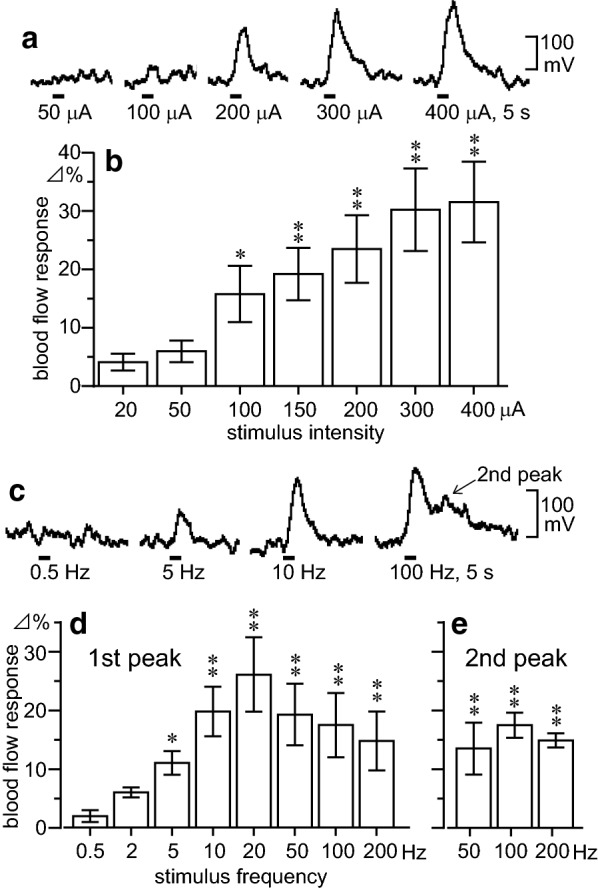
Olfactory bulb blood flow responses following olfactory nerve stimulation. The effects of various intensities (**a**, **b**) and frequencies (**c**–**e**) of electrical stimulation of the unilateral olfactory nerve on regional blood flow in the olfactory bulb ipsilateral to the stimulus. **a**, **b** Stimulus frequency, 20 Hz. **c**–**e** Stimulus intensity, 300 μA. **a**, **c** Sample recordings of regional blood flow in the olfactory bulb. **b**, **d**, **e** Summarized responses of regional blood flow in the olfactory bulb measured at the maximal level within 8 s (1st peak) or later (2nd peak) after the onset of stimulation, and expressed as the percentage increase from the pre-stimulus basal blood flow (mean value of the 10-s duration). Each column and vertical bar represents the mean ± SEM (*n* = 6). The data depict blood flow changes measured by laser Doppler (*n* = 4) or laser speckle (*n* = 2) flowmetry. *p < 0.05, **p < 0.01: significantly different from the responses at an intensity of 20 μA (**b**) or frequency of 0.5 Hz (**d**), using one-way repeated-measures ANOVA followed by Dunnett’s multiple comparison test

The effects of electrical stimulation of the unilateral olfactory nerve with different stimulus intensities (Fig. 1a, b) and frequencies (Fig. 1c–e) on regional blood flow in the olfactory bulb ipsilateral to the stimulation were examined.

When the olfactory nerve was electrically stimulated with various stimulus intensities at a constant frequency of 20 Hz, stimulation at intensities of ≥ 100 μA clearly produced a current-dependent increase in the blood flow in the olfactory bulb, as shown in sample recordings in Fig. 1a. The blood flow in the olfactory bulb usually started to increase approximately 1 s after the onset of stimulation and peaked near the end of the 5-s stimulation. The peak (maximum) responses of olfactory bulb blood flow following stimulation of the olfactory nerve with different intensities in six rats are summarized in Fig. 1b. Olfactory bulb blood flow significantly increased in response to stimulus intensities of ≥ 100 μA. The responses were largest to intensities of 300 and 400 μA. With a stimulus intensity of 300 μA, the blood flow reached its maximum of 30 ± 7% (range 17–53%). We used a maximal intensity of 300 μA for olfactory nerve stimulation in the subsequent experiments.

Figure 1c–e presents sample recordings and summarized graphs (*n* = 6 rats) of olfactory bulb blood flow responses to olfactory nerve stimulation with various stimulus frequencies at a constant intensity of 300 μA. Olfactory bulb blood flow was significantly increased by stimulation with frequencies of ≥ 5–200 Hz (Fig. 1c). The response was largest at a frequency of 20 Hz, reaching 26 ± 6% (range 11–55%; Fig. 1d). Stimulation with higher frequencies of 50–200 Hz produced a second peak blood flow response approximately 14–29 s after the onset of olfactory nerve stimulation (Fig. 1c, e).

### Spatiotemporal changes in cerebral blood flow following olfactory nerve stimulation

**Fig. 2:**
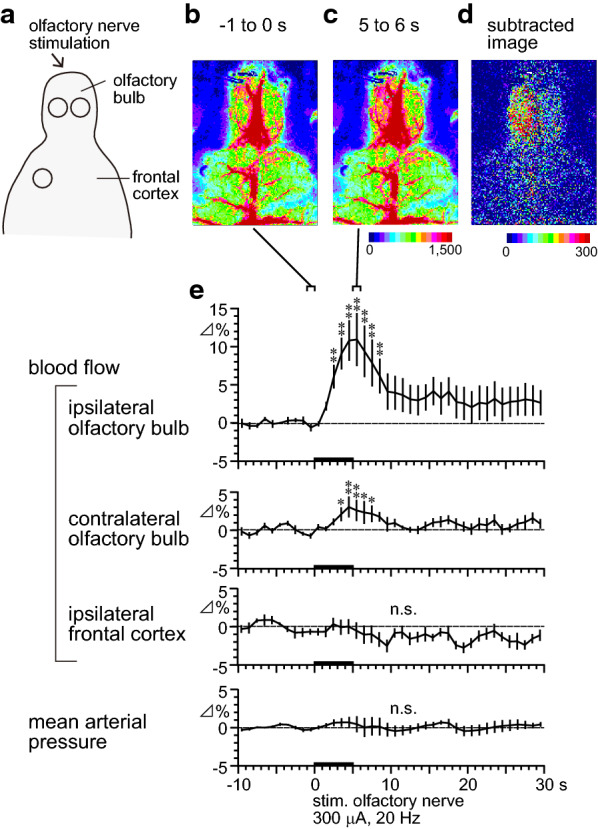
Spatiotemporal changes in regional cerebral blood flow following olfactory nerve stimulation. **a** Schema showing the area of blood flow recorded using a laser speckle contrast imager. **b**–**d** Sample recording of regional cerebral blood flow in one rat. **b**, **c** Averaged signal over selected periods of 1 s. **d** Differential signal changes obtained from **b** and **c** when subtracting the baseline (**b** −1 to 0 s) signal from the subsequent image (**c** 5–6 s). **e** Percentage signal changes in the ipsilateral and contralateral olfactory bulb and frontal cortex in response to olfactory nerve stimulation (data extracted from the region of interest indicated by the black circle in **a**) and mean arterial pressure averaged every 1 s. Changes in each parameter are expressed as the percentages of corresponding basal values (mean value for 10 s before time zero). Each point and vertical bar represent the mean ± SEM (*n* = 6). *p < 0.05, **p < 0.01: significantly different from the pre-stimulus basal values (−1 to 0 s) using one-way repeated-measures ANOVA followed by Dunnett’s multiple comparison test

Figure 2b–d presents the laser speckle flow images in a rat before and after olfactory nerve stimulation at 300 μA and 20 Hz for 5 s. Electrical stimulation of the olfactory nerve produced a marked increase in blood flow in the olfactory bulb ipsilateral to the stimulus at 5–6 s after the onset of stimulation. By contrast, the stimulation produced an extremely slight increase in blood flow in the olfactory bulb contralateral to the stimulus. Blood flow in the bilateral frontal cortices was not affected by the stimulation.

Figure 2e summarizes the time courses of the responses of regional blood flow in the ipsilateral and contralateral olfactory bulb and ipsilateral frontal cortex to olfactory nerve stimulation, and mean arterial pressure measured every 1 s in six rats. Blood flow in the olfactory bulb ipsilateral to the olfactory nerve stimulation was significantly increased by the stimulation. After the onset of stimulation, a significant increase in blood flow appeared at 2–3 s and peaked near the end of the stimulation period, and after which blood flow gradually returned to the pre-stimulus basal level after the cessation of stimulation. The maximum change of blood flow in the ipsilateral olfactory bulb was 11 ± 3% (range 3–26%) at 5–6 s. Blood flow in the olfactory bulb contralateral to the olfactory nerve stimulation was slightly increased following the stimulation. The maximum change of blood flow in the contralateral olfactory bulb was 3 ± 1% at 4–5 s. Moreover, blood flow in the frontal cortex ipsilateral to the olfactory nerve stimulation and mean arterial pressure exhibited no obvious changes following olfactory nerve stimulation.

From these results, we focused on blood flow in the olfactory bulb ipsilateral to the olfactory nerve stimulation, and the effect of nicotine on the response was examined.

### Effect of intravenous nicotine on the olfactory bulb blood flow response induced by olfactory nerve stimulation

We chose three stimulus frequencies (2, 20, and 100 Hz) at a constant intensity of 300 μA to analyze the effect of nicotine on the olfactory bulb blood flow response. Before the nicotine injection, the olfactory bulb blood flow responses elicited by olfactory nerve stimulation using three different frequencies were confirmed to be stable between two trials.

**Fig. 3:**
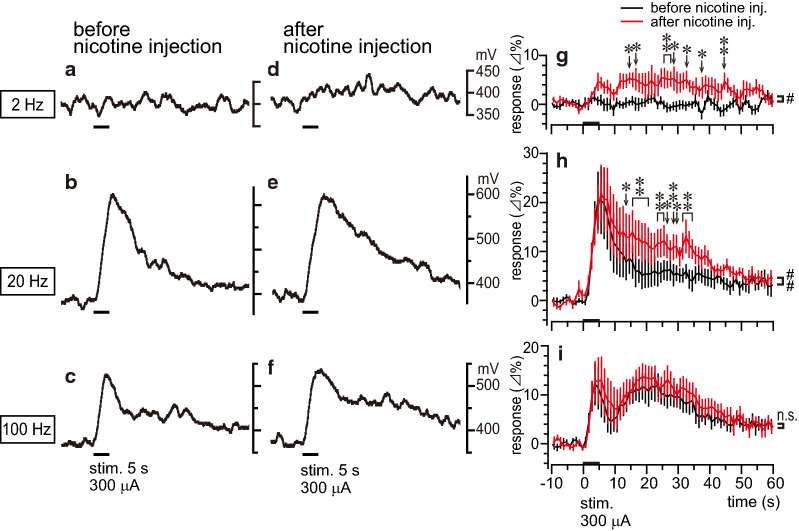
Effect of nicotine on olfactory bulb blood flow responses induced by olfactory nerve stimulation. Stimulus frequencies were 2 (**a**, **d**, **g**), 20 (**b**, **e**, **h**), and 100 Hz (**c**, **f**, **i**). **a**–**f** Sample recordings taken before (**a**–**c**) and after (**d**–**f**) the intravenous injection of nicotine (30 μg/kg). **g**–**i** Graph summarizing the time course of regional blood flow responses in the olfactory bulb ipsilateral to olfactory nerve stimulation averaged every 1 s, before (black line) and after (red line) nicotine injection. Changes in regional blood flow are expressed as percentages of the corresponding basal values (mean value for 10 s before time zero). Each point and vertical bar represent the mean ± SEM (*n* = 8). Data depict blood flow changes measured using laser Doppler (*n* = 4) or laser speckle (*n* = 4) flowmetry. Statistical comparisons of changes in regional blood flow in response to olfactory nerve stimulation before and after nicotine injection were conducted using two-way repeated-measures ANOVA (^#^*p* < 0.05; ^##^*p* < 0.01) followed by Bonferroni’s multiple comparison test (*p < 0.05; **p < 0.01)

Figure 3a–f shows sample recordings of regional blood flow in the olfactory bulb ipsilateral to the olfactory nerve stimulation with three different frequencies, obtained before (Fig. 3a–c) and at 7–13 min after nicotine (30 μg/kg) administration (Fig. 3d–f) in a rat. Figure 3g–i summarizes the time course responses of olfactory bulb blood flow to olfactory nerve stimulation using these three frequencies obtained before and after (tested at 5–20 min) nicotine injection in eight rats. Stimulation with a frequency of 2 Hz produced no obvious changes in blood flow before the nicotine injection (Fig. 3a and black line in g), but it produced a significant increase in blood flow after the nicotine injection (*p* < 0.01, assessed using one-way repeated-measures ANOVA; Fig. 3d and red line in g). There was a significant difference in the olfactory bulb blood flow response to 2 Hz stimulation between these two conditions (before vs. after nicotine injection; *p* < 0.05, assessed using two-way repeated-measures ANOVA). Comparing the increase in olfactory bulb blood flow responses to 20-Hz stimulation before and after nicotine injection, the duration of increased blood flow following olfactory nerve stimulation was prolonged by nicotine administration (Fig. 3b, e, h). There was a significant difference in the olfactory bulb blood flow response to 20-Hz stimulation between these two conditions (before vs. after nicotine injection; *p* < 0.01, assessed using two-way repeated-measures ANOVA). The increase in olfactory bulb blood flow evoked by 100-Hz stimulation tended to become larger after the nicotine injection, but there was no statistical difference in blood flow responses between the two conditions (before vs. after nicotine injection; Fig. 3c, f, i). The basal level of blood flow in the olfactory bulb and mean arterial pressure were not changed significantly by nicotine administration (30 μg/kg). In more detail, the averaged values for 10 s just before stimulation at 20 Hz were compared before and after nicotine injection. Olfactory bulb blood flow before injection was 100% vs. 102 ± 1% after, and mean arterial pressure before injection 77 ± 3 mmHg vs. 73 ± 3 mmHg after.

**Fig. 4:**
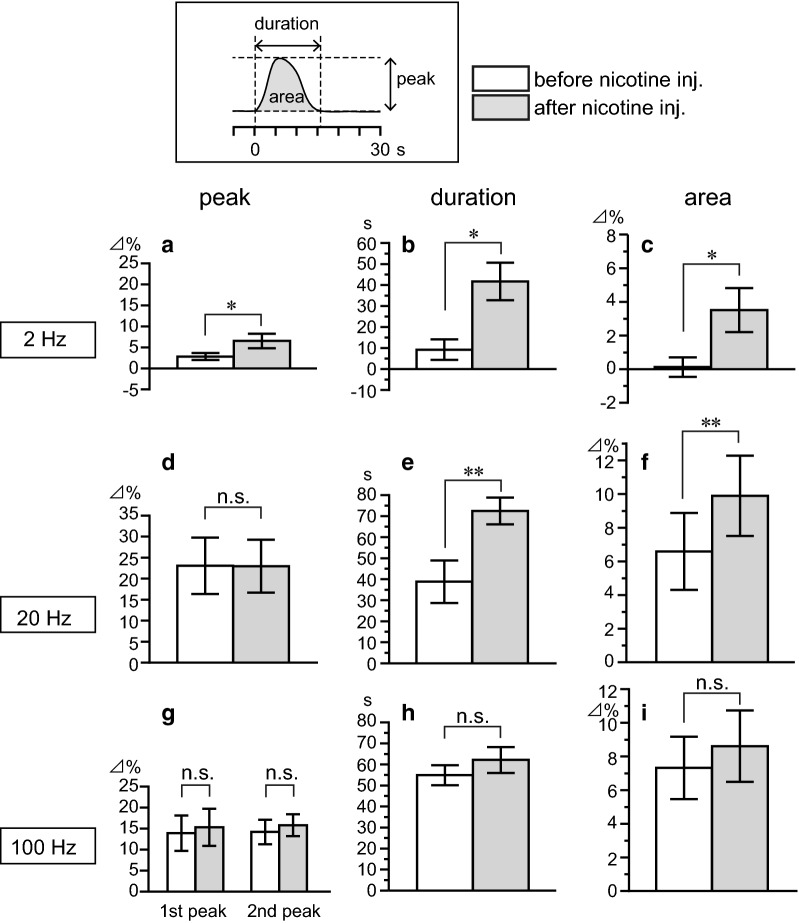
Summary of nicotine effects on olfactory bulb blood flow responses induced by olfactory nerve stimulation. The stimulus frequencies were 2 (**a**–**c**), 20 (**d**–**f**), and 100 Hz (**g**–**i**). **a**, **d**, **g** Peak responses were measured by maximum blood flow changes within 8 s (1st peak) or later (2nd peak) after the onset of stimulation (see upper inset), and expressed as percentages of the corresponding basal values (mean value for 10 s before time zero). **b**, **e**, **h** Durations were measured by time (s) until the increased blood flow returned to the basal values (see upper inset). **c**, **f**, **i** Area under the curve (shaded gray shown in the upper inset) were measured as averaged blood flow values for a 60-s period after the onset of stimulation and expressed as percentages of the corresponding basal values (mean value for 10 s before time zero). Each column and vertical bar represent the mean ± SEM (*n* = 8). *p < 0.05, **p < 0.01: significantly different from the response before nicotine injection as determined using Wilcoxon’s matched-pairs signed-rank test

Using the same data shown in Fig. 3, the peak response (%), duration of response (s), and 60-s area (%) for olfactory bulb blood flow response following stimulation of the olfactory nerve using three different frequencies were analyzed and compared between before and after nicotine injection (Fig. 4). Concerning the olfactory bulb blood flow responses to 2-Hz stimulation, nicotine significantly augmented the peak response (Fig. 4a), the duration of response (Fig. 4b), and the area response (Fig. 4c). Concerning the olfactory bulb blood flow responses to 20-Hz stimulation, nicotine did not change the peak response (Fig. 4d), while it significantly prolonged the duration of response (Fig. 4e), thereby significantly augmented the 60-s area response by approximately 1.5-fold (Fig. 4f). Conversely, olfactory bulb blood flow responses to 100 Hz stimulation were not significantly changed by nicotine injection in any analyses (Fig. 4g–i).

The augmented olfactory bulb blood flow responses to 2- and 20-Hz stimulation obtained at 5–20 min after the injection of nicotine had dissipated, when measured at 30–90 min after nicotine injection in four rats tested.

## Discussion

Our previous study reported that activation of α4β2-like subtype nAChRs in the brain by the intravenous injection of nicotine potentiates the odor stimulation (5% amyl acetate)-induced increase in olfactory bulb blood flow response in anesthetized rats [15]. In the present study, we first demonstrated that electrical stimulation of the olfactory nerve instead of odor stimulation increased blood flow in the olfactory bulb. Furthermore, we found that nAChR activation via the intravenous injection of nicotine potentiates those olfactory bulb blood flow responses. This study found that the nicotine-induced potentiation of olfactory bulb blood flow responses occurred when the olfactory nerve was stimulated at frequencies of 2 and 20 Hz, but not 100 Hz. The present findings provide additional evidence that nAChR activation potentiates olfactory bulb blood flow responses to olfactory input, and the potentiation occurs when the strength of the input is weak and intermediate, but not strong. Our new findings, which used olfactory nerve stimulation instead of natural olfactory stimulation by odor inhalation, provide stronger neurophysiological evidence that potentiation of olfactory input-induced increase in olfactory bulb blood flow by nAChR activation occurs at the level of the olfactory bulb.

In the olfactory system, olfactory sensory neurons (olfactory nerve) in the olfactory epithelium detect odorants and send information about the sense of smell to the olfactory bulb. The olfactory nerve was spontaneously active at approximately 1.2 Hz in an olfactory nerve recording study conducted in anesthetized rats [17]. The firing rate of the olfactory nerve increased with increasing odor concentration, reaching approximately 86 Hz in an in vivo olfactory nerve recording study in rats [17], or even a higher frequency of 200–300 Hz in isolated mouse olfactory sensory neurons [18]. In the present study, before the injection of nicotine, olfactory nerve stimulation (at an intensity of 300 μA) with frequencies of ≥ 5 Hz produced an increase in blood flow in the olfactory bulb ipsilateral to the nerve stimulation, peaking near the end of the 5-s stimulation. Stimulation of the olfactory nerve at higher frequencies of 50–200 Hz produced a second peak blood flow response approximately 14–29 s after the onset of the stimulation.

It is well established that odor stimulation increases regional blood flow in the rodent olfactory bulb, in association with neuronal activities [11–14]. A recent study by Boido et al. [10] revealed a linear relationship between vascular responses in the olfactory bulb to odors and Ca^2+^ signals from local (principal) neurons, i.e., mitral cells and external tufted cells, in an imaging study in the same mouse and suggested that blood flow in the olfactory bulb can be used as a quantitative marker of synaptic activation. In the present study, the observed increase in olfactory blood flow in response to olfactory nerve stimulation may reflect synaptic activation in the olfactory bulb. In olfactory bulb principal neurons, external tufted cells exhibit a lower threshold (higher sensitivity) for excitation compared with mitral cells in response to olfactory sensory inputs [29–31]. In response to high-frequency (50 Hz) olfactory nerve stimulation, mitral cells produce sustained responses, whereas external tufted cells respond transiently [32]. We therefore speculated that the first peak olfactory bulb blood flow response evoked by olfactory nerve stimulation may reflect external tufted cell excitation, whereas the second peak olfactory bulb blood flow response evoked by high-frequency olfactory nerve stimulation may largely reflect mitral cell excitation.

In the present study, the magnitude of the first peak blood flow response was largest at 20 Hz of stimulation and became smaller at higher frequencies (50–200 Hz). Mechanism of this attenuation by high-frequency stimulation is not clear. The olfactory nerve consists of unmyelinated axons [33, 34] with a variety of diameters (50–400 nm) [33]. High-frequency stimulation may release other vasoactive transmitters [35] in addition to glutamate from different types of olfactory nerve fibers.

The ethmoidal nerve, a branch of the ophthalmic division of the trigeminal nerve, innervates both the olfactory epithelium and the olfactory bulb [36, 37] and detects odors—strong irritating odors in particular [38]. In the present study, it is possible that electrical stimulation applied to the olfactory nerve also stimulated nearby ethmoidal nerve. However, electrical stimulation of a sub-branch of the trigeminal nerve (the nasociliary nerve, which contains ethmoidal nerve) reportedly increased regional cortical blood flow in anesthetized rats [39]. In our study, olfactory nerve stimulation produced an increase in olfactory bulb blood flow, but did not change frontal cortical blood flow. We therefore speculated that the ethmoidal nerve is less likely to be stimulated by olfactory nerve stimulation procedure.

The present study demonstrated that the intravenous injection of nicotine (30 μg/kg) potentiated olfactory bulb blood flow responses to olfactory nerve stimulation at frequencies of 2 and 20 Hz, but not 100 Hz. These results suggest that the nicotine-induced potentiation of olfactory bulb responses occurs when the strength of the olfactory input is weak and intermediate, but not strong. In a Ca^2+^ imaging study using the mouse olfactory bulb, Bendahmane et al. [40] indicated that electrical stimulation of the horizontal limb of the diagonal band of Broca (HDB), which sends cholinergic fibers to the olfactory bulb [41–43], leads to the activity-dependent modulation of glomerular odor responses, whereby weak-to-moderate responses are enhanced and strong responses are reduced. Our present results of nicotine-induced potentiation of olfactory bulb blood flow responses to olfactory nerve stimulation with 2 and 20 Hz, but not with 100 Hz, more or less agree with the results of Bendahmane et al. [40].

Concerning the statistical result of no significant effect of nicotine on olfactory bulb blood flow response to 100 Hz stimulation (e.g., Fig. 4i, *n* = 8, *p* = 0.38, effect size = 0.38), a type II error (false negatives) is a possibility due to the small number of animals. Several studies have reported that a physiologically appropriate firing rate of olfactory nerve in response to odor is less than approximately 50 Hz [32, 44, 45]. Therefore, in this study we did not increase the number of animals subjected to 100 Hz of stimulation for ethical reasons. When designing the protocol of the present experiment, the estimated sample size was based on our previous study of the effect nicotine injection (30 μg/kg) on odor-induced olfactory bulb blood flow response, which increased by 5.9 ± 3.9% (mean ± standard deviation) [15]. With 90% power and a two-sided significance level of 5%, the required sample size was 8, as determined by software calculations (G* Power 3.1 [46]). Our decision to test 8 rats was therefore appropriate.

Cholinergic neurons in the basal forebrain including the HDB, as well as the olfactory bulb express the mRNA of multiple subtypes of nAChRs, including the alpha 4 and beta 2 subunits [47–49]. Therefore, we can expect that the nicotine-induced potentiation of olfactory bulb blood flow responses may occur through the activation of nAChRs in cholinergic neurons in the HDB and/or olfactory bulb. Further study is needed to explore the location of nAChRs contributing to the potentiation of olfactory bulb blood flow responses.

Our previous study reported that HDB stimulation increased ACh release, but did not change blood flow in the olfactory bulb [9]. Without odor input, olfactory bulb neurons (principal neurons, i.e., mitral cells and external tufted cells) were not influenced by HDB cholinergic activation as measured by Ca^2+^ signals [40]. This may explain our previous finding that HDB cholinergic activation did not change blood flow in the olfactory bulb [9]. Our present study found that nAChRs activation potentiates olfactory bulb blood flow in response to olfactory nerve stimulation. We speculated that cholinergic inputs to the olfactory bulb do not directly influence neuronal activity at rest, but potentiate synaptic transmission evoked by olfactory inputs [40], and thereby increase the olfactory bulb blood flow response.

Olfactory function is known to decline in the early stage of Alzheimer’s disease [50, 51]. In patients with Alzheimer’s disease, atrophy of the basal forebrain cholinergic system [52, 53], as well as reduced α4β2-nAChR availability, especially within the basal forebrain, frontotemporal cortices, and hippocampus [54] has been reported even in the early stage of the disease. Therefore, we can speculate that the functional decline of nicotinic cholinergic transmission as well as the degeneration of basal forebrain cholinergic neurons may be a causative factor for the decline of olfactory function in the early stage of Alzheimer’s disease.

Concerning the physiological roles, the nicotine-induced potentiation of increase in olfactory bulb blood flow in response to olfactory input may contribute to the neuronal turnover in the olfactory bulb and short-term olfactory memory [55–57], thereby maintaining olfactory function and cognitive function.

## Conclusion

In conclusion, our present study demonstrated that direct electrical stimulation of the olfactory nerve instead of odor stimulation induced an increase in olfactory bulb blood flow. Furthermore, this study found that the activation of nAChRs by intravenous nicotine potentiates the olfactory bulb blood flow response to olfactory input of weak and intermediate intensity. These results prove important evidences of olfactory bulb blood flow response by olfactory nerve stimulation, and of its potentiation through activation of nicotinic cholinergic transmission.

## Data Availability

The data that support the findings of this study are available from the corresponding author on reasonable request.

## Abbreviation

nAChR: Nicotinic acetylcholine receptor
